# Atypical hand-foot-mouth disease in children: a hospital-based prospective cohort study

**DOI:** 10.1186/1743-422X-10-209

**Published:** 2013-06-24

**Authors:** Wen-Chan Huang, Li-Min Huang, Chun-Yi Lu, Ai-Ling Cheng, Luan-Yin Chang

**Affiliations:** 1 Department of Pediatrics, National Taiwan University Hospital, College of Medicine, National Taiwan University, Taipei, Taiwan

**Keywords:** Hand-foot-mouth disease, Onychomadesis, Vesiculobullous rash, Large vesicles, Enterovirus, Pigmentation, Phylogenetic analysis

## Abstract

**Background:**

In 2010, we observed children with atypical presentations of hand-foot-mouth disease (HFMD), such as rashes on earlobes and faces, or bullae on trunks and bilateral limbs. Hyperpigmentation later developed as the bullous lesions crusted. Thus, we intended to study the etiology of the illness and the phylogeny of the pathogens.

**Method:**

Patients were prospectively enrolled in a tertiary medical center in Taipei, Taiwan. The definition of atypical HFMD includes symptoms of acute viral infection with either of the following presentations: (1) maculopapular rashes presenting on the trunks, buttocks or facial areas, or (2) large vesicles or bullae on any sites of the body. Patients were classified into two groups according to vesicle sizes by two pediatricians at different points in time. The large vesicle group was defined as having vesciculobullous lesions ≥ 1 cm in diameter; the small rashes group had maculopapular rashes < 1cm in diameter. Two throat swabs were collected from each patient for virus isolation and reverse transcription polymerase chain reactions.

**Results:**

We enrolled 101 patients between March and December 2010. The mean age of the participants was 3.3 ± 3.0 years (median age: 2.5 years, range: 21 days-13.5 years). The ratio of males to females was 1.8 to 1. All samples were enterovirus-positive, including coxsackievirus A6 (80%), coxsackievirus A16 (6%), enterovirus 71 (1%), coxsackievirus A5 (1%) and 12 non-typable enterovirus (12%). Bullous fluid aspirated from 2 patients also grew coxsackievirus A6. Among the patients infected with coxsackievirus A6, 54% (45/81) had bullae, compared to 25% (5/20) of those having non-coxsackievirus A6 infections (*P*=0.02). Fourteen cases had myoclonic jerks and one boy was diagnosed with febrile convulsions. None had complications or sequelae. Phylogenetic analysis showed the strains in Taiwan in 2010 shared more commonality with strains from Finland in 2009 (GenBank: FJ870502-FJ870508), and were close to those circulating in Japan in 2011 (GenBank: AB649286-AB649291).

**Conclusions:**

Coxsackievirus A6 infections may cause atypical manifestations of HFMD, including vesicles or papules on faces or bullae on trunks. These features could provide valuable information to distinguish this versatile enterovirus infection from other virus-induced vesiculobullous diseases.

## Background

In children with infectious diseases, cutaneous lesions usually provide clues for early diagnoses [1,2]. Hand-foot-mouth disease (HFMD), a common and potentially fatal infectious disease in children, largely relies on clinical manifestations for early diagnosis, including maculopapular or vesicular rashes on soles, palms and buttocks, and oral ulcers in the pharynx [3]. Enteroviruses, particularly enterovirus 71 (EV71) and coxsackievirus A16 (CVA16), are known to cause HFMD. Most oral-dermatological phenotypes are self-limited; however, in certain patients, EV 71 infection may lead to severe neurological complications, especially in young children [4]. For example, during an outbreak in 1998 in Taiwan, EV71 caused 78 deaths out of more than 129,000 patients who had presented with HFMD or herpangina initially [5]. Since then, the Centers for Disease Control in Taiwan (CDC-Taiwan) have been closely monitoring the trends of enterovirus infections by cooperating with the Sentinel Physicians Surveillance and Virology Reference Laboratories Network. The surveillance database is published weekly and provides information regarding the circulating trends of enteroviruses [6].

Since spring 2010, we have observed an unusual type of skin lesion presenting on children in Taiwan, including large vesicles or bullae over the limbs, trunks or buttocks and papules on faces, accompanied by fevers, stomatitis, and sore throats. Moreover, a few children developed onychomadesis, desquamation and skin pigmentation over the areas previously presenting with large vesicles or bullae. These presentations shared several of the traits of HFMD, including vesicular regions on limbs and oral ulcers, yet with distinct vesiculobullous lesions from the typical maculopapular rashes. Over the past decade, the association between onychomadesis and enterovirus infection has been reported in coxsackievirus A5 (CVA5), coxsackievirus A6 (CVA6) and coxsackievirus A10 [7,8]. However, whether the morphology and distribution of vesicles and accompanying symptoms varied among enterovirus infections of different serotypes remained unknown. Therefore, we aimed to evaluate the etiology of the atypical presentations of HFMD during the epidemic period.

## Results

### Demography

**Figure 1:**
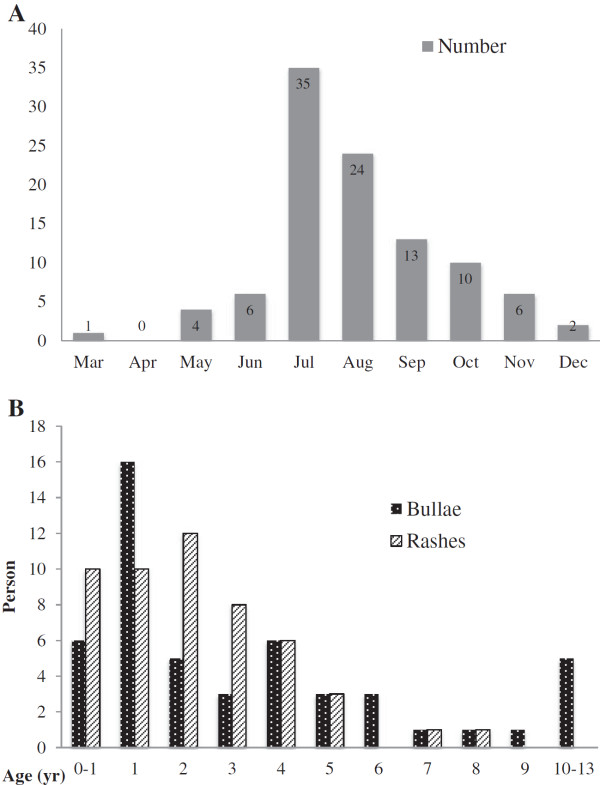
**Monthly and age distribution of atypical HFMD. (A)** This epidemic of CVA6 started in early spring of 2010. The enrollment of patients peaked in between July and October. **(B)** Most of the children in the small rash group were younger than 6 year-old. In the bullae group, 78% were children under 5 year of age and 10% were teens.

Out of the 156 children screened between March and December 2010, 101 children were enrolled in the study. Most of them were recruited in the summer (Figure 1). Fifty-five children were excluded because of incomplete demographic data or lack of adequate clinical samples. All participants had unremarkable medical histories. Eighty percent (81/101) of the cases were recruited from outpatient clinics, and the rest (20/101) from the emergency department. Contact history was reported in 34% (34/101) of the participants, five at school and 29 in the household. The mean age of the participants was 3.3 ± 3.0 years (median: 2.5 years, range: 21 days-13.5 years). Males outnumbered female by 1.8 to 1, especially in patients with large bullae (p=0.004).

### Virology examinations

The yield rate of viral cultures was only 18% (18/101). Six specimens were CVA16-positive and 1 was EV71-positive. RT-PCR was performed for these 7 specimens and confirmed the same results of the viral cultures. Four were positive for enterovirus with uncertain serotypes due to an initial limitation of available antibodies, and were later confirmed to be CVA6 by RT-PCR and VP1 sequencing. Of the remaining seven samples, one was echovirus 9, five were cytomegalovirus, and the last was herpes simplex virus. Cytomegalovirus and herpes simplex virus were considered latent viruses and not related to atypical HFMD.

Throat swabs of all participants were shown to be enterovirus-positive by RT-PCR and the subsequent VP1 sequencing revealed eighty-one (80%) to be the serotype of CVA6, six (6%) of CVA16, one (1%) of EV71, one (1%) of CVA5 and 12 (12%) of non-typable EV (NTEV), which could not be further serotyped due to low viral titers. Additional samples from bullous fluid were collected from 2 patients, which were further detected with RT-PCR and VP1 sequencing. Both were positive for CVA6. The viral load was 1.5*10^5^ copies/mL from Patient 1 (12.5 years old, male) and 1.9*10^7^ copies/mL from Patient 2 (2.9 years old, male). The viral loads of the throat samples, from these two children on the same dates as the fluid sample being collected, were lower than that of the bulla fluid (Patient 1: 1*10^3^ copies/mL; Patient 2: 5*10^5^ copies/mL).

### Clinical presentations

**Figure 2:**
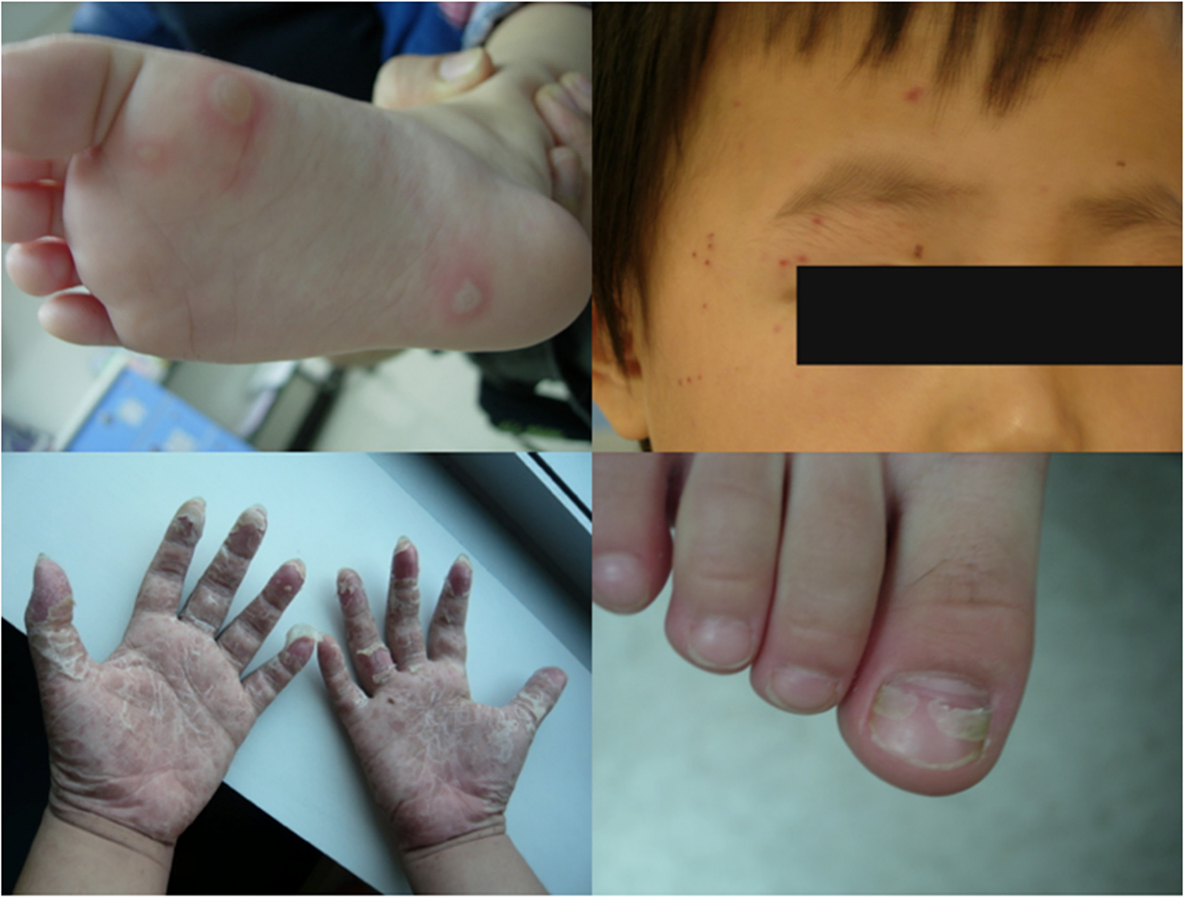
**Dermatologic presentations of atypical HFMD.** Erythema was frequently observed at the base of large vesicles. These lesions were distributed on limbs, trunks or perioral areas. (Top, left) Bullae were formed on a 5 year-old boy’s sole, which caused mild pruritus and prickling sensations. (Top, right) Atypical rashes of CVA6 spread on a 4 year-old child’s forehead and temporal area of the face. The lesions did not involve the cornea or conjunctiva. (Bottom, left) Late presentation of atypical HFMD, severe desquamation was presented on bilateral palms and soles of a 12 year-old boy. Ten days earlier, he had marked blisters on his bilateral limbs and scattered vesicles on his buttock, yet without fever or other systemic symptoms. (Bottom, right) Onychomadesis occurred on the 2 weeks post CVA6 infection.

Out of the 101 patients, the most common symptom was fever (80%), followed by sore throats (59%), itchy skin (32%), upper airway symptoms (27%), vomiting/diarrhea (17%), and myalgia (2%). Neurological symptoms occurred in 15 children, including myoclonic jerks (14%, 14/101) and febrile seizures (1%, 1/101). For the dermatologic presentations, most of the children with atypical HFMD presented lesions on their limbs (knee and/or elbows, 98%), the typical sites of HFMD. Among the atypical areas, lesions were most commonly distributed on faces (41%), along with buttocks (31%) and trunks (29%). Vesiculobullous eruptions could also be observed on thighs, on which rarely developed lesions in typical HFMD. The patterns of the phenotype of atypical HFMD are shown in Figure 2.

The analysis of clinical presentations between two groups of skin lesions and between CVA6 and non-CVA6 infected patients are listed in Tables 1 and 2, respectively. Children with large vesicles were more frequently CVA6-positive (large vesicle group vs. small rash group: 90% vs. 70%, P=0.01) and this type of vesiculobullous lesion more commonly presented on trunks in the large vesicle group (44% vs. 16% P=0.004). Also, children infected with CVA6 had higher chances of developing large vesicles than those without (56% vs. 25%, *P*=0.02). Pruritus was the most common complaint, and appeared more frequently in the large vesicles group (44%) than in the small rashes group (20%, *P*=0.01). The incidences of neurological symptoms were similar between two skin lesion groups and between two groups of CVA6 and non-CVA6 infections. None of the patients had complications or sequelae.

**Table 1:** Clinical presentations of two types of atypical HFMD

	**A typical HFMD (N=101)**	**Large vesicles (N=50)**	**Small rashes (N=51)**	P value*
**Demographics**				
Age (year)	3.3 (± 3.8)	4.0 (± 3.7)	2.7 (± 1.8)	0.12
Male	65 (64%)	35 (70%)	30 (59%)	**0.004**
Female	36 (36%)	15 (30%)	21 (41%)	
**Etiology**				
CVA6	81 (80%)	45 (90%)	36 (70%)	**0.01**
**Sites**				
Limbs	98 (97%)	49 (98%)	49 (96%)	1.00
Arm/Leg/Thigh	71 (70%)	35 (70%)	36 (70%)	1.00
Trunk	29 (29%)	21(42%)	8 (16%)	**0.004**
Buttocks	31 (31%)	17 (34%)	14 (27%)	0.52
Face	40 (41%)	22 (44%)	18 (35%)	0.42
Ear lobes	11 (11%)	6 (12%)	5 (10%)	0.76
Perioral area	26 (26%)	16 (32%)	10 (20%)	0.18
**Symptoms**				
Fever	80 (80%)	40 (80%)	39 (76%)	0.81
Sore throat	59 (58%)	33 (66%)	26 (51%)	0.16
Cough/rhinorrhea	27 (27%)	14 (28%)	13 (25%)	0.83
Vomit/diarrhea	17 (17%)	6 (12%)	11 (22%)	0.29
Skin lesions				
Itchiness	32 (32%)	22 (44%)	10 (20%)	**0.01**
Desquamation	1 (1%)	1 (2%)	0 (0%)	0.50
Pigmentation	6 (9%)	5 (10%)	1 (2%)	0.11
Prickle	2 (2%)	2 (4%)	0 (0%)	0.24
Myoclonic jerks	14 (14%)	5 (10%)	9 (18%)	0.39
Myalgia	2 (2%)	2 (4%)	0 (0%)	0.24

* *P* value represents the comparison of total numbers between male and female.

**Table 2:** Clinical presentations of patients acquiring CVA6 and non-CVA6 enterovirus infection

	**CVA6 positive (N=81)**	Non-CVA6* (N=20)	**P value**
**Demographics**			
Age (year)	3.5 (±3.2)	2.8 (±1.5)	0.92
Male	54 (67%)	11 (55%)	0.33
Female	27 (33%)	9 (45%)	
**Sites**			
Limbs	80 (99%)	18 (90%)	0.10
Arm/Leg/Thigh	56 (69%)	16 (80%)	0.79
Trunk	22 (27%)	7 (35%)	0.58
Buttocks	25 (30%)	6 (30%)	1.00
Face	34 (41%)	8 (40%)	0.80
Ear lobes	11 (13%)	0	0.12
Perioral area	23 (28%)	4 (20%)	0.27
**Morphology**			
Large vesicles	45 (56%)	5 (25%)	**0.02**
**Symptoms**			
Fever	66 (80%)	15 (75%)	0.37
Sore throat	49 (60%)	10 (50%)	0.45
Cough/rhinorrhea	23 (28%)	7 (35%)	0.58
Vomit/diarrhea	11 (13%)	6 (30%)	0.10
Skin lesions			
Itchiness	27 (33%)	5 (25%)	0.60
Pigmentation	6 (7%)	3 (15%)	0.34
Prickle	2 (2%)	0 (0%)	1.00
Myoclonic jerks	11 (13%)	3 (15%)	1.00
**Hospitalization**			
Days (days)	3.4 (± 1.3)	2.3 (± 0.6)	0.24
Age (years)	2.3 (± 3.1)	2.3 (± 0.6)	0.30

CVA6 denotes coxsackievirus A6.

* including 6 of CVA16, 1 of EV71, 1 of CVA5 and 12 of non-typable enterovirus.

### Hospitalization

Seventeen cases (17%) were hospitalized. All were discharged smoothly. Most hospitalized patients were preschool children (mean age: 2.6 ± 2.9 years) and the mean hospitalization duration was 3.2 ± 1.3 days. Dehydration was the primary cause for hospitalization (76%). Among 15 children presenting neurological symptoms, only two patients required hospitalized evaluation. One child developed frequent myoclonic jerks and the other had febrile convulsions. The hospitalization rate did not differ between two skin-lesion groups, or between CVA6 and non-CVA6 groups.

### Sequence analysis

**Figure 3:**
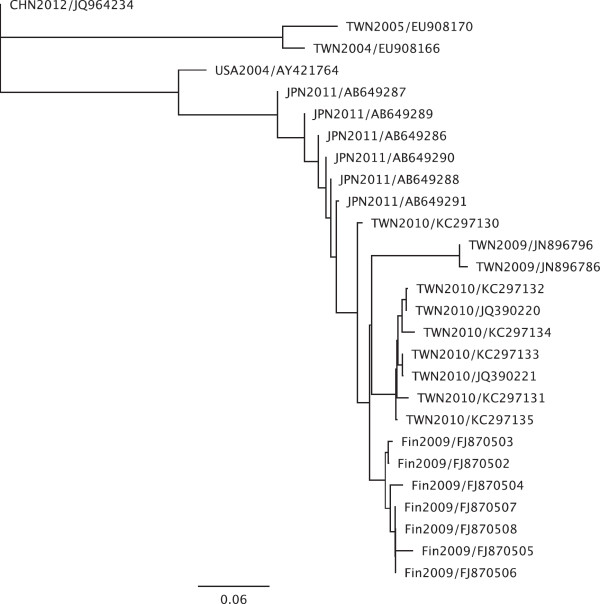
**The analysis of phylogeny is based on partial VP1 gene sequence (264–377 nucleotides).** Clade I, is CVA6 strain HN421 (JQ964234), isolated from Henan, China in 2012. Clade II are the two circulating strains of CVA6 in Taiwan between 2004 and 2005 (EU908166, EU908170). Clade III includes the prototype CVA6 strain from the USA (AY421764), Japanese circulating strains in 2011 (AB649286- AB649291), 2009 Finland strains (FJ870502- FJ870508) and Taiwanese circulating strains in 2009–2010 (JN896786, JN896796, JQ390220, JQ390221) and the strains of the current study in 2010 (KC297130-KC297135).

Based on the sequences of the partial coding sequence of the VP1 gene (GenBank: KC297130-KC297135), the consensus sequences of our current study shared 93.1%-95.8% nucleotide identity (100% amino acid identity) with the 2009 Finland strain (GenBank: FJ870502- FJ870508) and were also close to those circulating in Japan in 2011 (GenBank: AB649286-AB649291) (Figure 3).

## Discussion

Our data showed most of the CVA6 infections are a common cause of atypical HFMD in children with relatively benign courses. Among the lesions of atypical HFMD, distinct bullae or large vesicles were more likely to occur on CVA6-positive children. Also, children with CVA6 infections had higher chances developing bullous vesicles.

Before the epidemic in 2010, CVA6 circulation had been observed in 2007 and 2009 in Northern Taiwan. In the study by Lo et al. [9], most children infected with CVA6 were < 7 years of age. Although 2% of the children (3/141) had meningitis or encephalitis, none had sequelae. The frequencies of myoclonic jerks (21.3%) and febrile convulsions (3.5%) reported in Lo et al’s study were similar to ours. However, less than 13% of children presented with HFMD. Herpangina was the predominant phenotype, which might be accompanied by fever, decreased oral intake and upper respiratory symptoms (e.g. cough and rhinorrhea). The epidemic CVA6 strain in the current study in 2010 shared a great deal of genetic commonality with the CVA6 strain in 2009 in Taiwan as determined by VP1 sequencing. Chen et al. sequenced complete genomes of circulating CVA6 strains between 2009 and 2010 in Taiwan, and revealed little difference in VP1 sequences. However, several changes in amino acids were noted in structure proteins (VP2 and VP3) and non-structure proteins (2A, 3C and 3D) [10]. Whether the variations in amino acids contribute to the discerning clinical presentations in 2010 still needs further investigation.

In our study, CVA6 was the major strain of enterovirus that caused atypical HFMD. In the same epidemic period, CDC-Taiwan retrospectively analyzed 130 patients with laboratory-confirmed CVA6 infections. Their findings showed that CVA6 patients had higher chances of experiencing desquamation (51%) and onychomadesis (37%) [11]. This study did not analyze early presentations of the infection as in ours. Thus, the two studies are in some part complementary, showing that in CVA6 infection, vesiculobullous lesions might develop early, followed by marked desquamation and pigmentation on palms and soles as the bullae crusted. Nail deformities could also be observed after the resolution of infection. These uncommon manifestations may provide valuable information to detect enterovirus infections in endemic countries. Early diagnosis is beneficial to pediatric patients in order to limit invasive examinations, such as skin biopsies, when determining the pathogenesis.

We examined the bullous fluid collected from the skin lesions. The viral loads of CVA6 were higher in the bullous fluid than in the throat swabs. Similar observations have not been reported among the other HFMD patients. Vesicular or bullous fluid may be considered a specimen for a more sensitive but also more specific source to determine the infectious etiology of HFMD, since issues may be raised that bystander viruses and true pathogens are indistinguishable in samples from throats and feces. In murine models, viral titers of EV71 were much higher in muscle and skin compared to those in the intestines or brain [12], suggesting enteroviruses also propagate in other organs. It once again addresses the importance of hand hygiene in the prevention of HFMD. The transmission of enterovirus is not limited to the oral-fecal route. Rather, it may pass on to other hosts through fluid from ruptured skin lesions.

In the summer of 2008, a CVA6 outbreak was reported in Finland and Spain, and the circulating strains in these two countries shared high levels of similarity based on partial VP1 sequences [7,8]. In the following years, outbreaks of vesicular-bullous eruptions and onychomadesis occurred in Taiwan (2010), Japan (2011) and the United States (2012), and CVA6 was the viral stain responsible for the events [11,13-15]. According to these reports and ours, the majority of the infected patients had uncomplicated symptoms. The clinical presentations of CVA6 infections were quite different from those of EV71-infected children, who usually presented small papules or vesicles on distal limbs. Also, young children with EV71 infections had much higher chances of developing severe neurological complications, sequalae or mortalities [16]. Moreover, CVA6 has become one of the endemic strains of enterovirus in Singapore, China and Thailand in recent years [17-19]. It is not known whether CVA6 may transform into a more virulent stain as it circulates in broader regions. Further research is needed to understand the mechanisms that cause atypical HFMD and in monitoring CVA6 epidemiology.

Several limitations of this study are summarized: (1) children with maculopapular rashes over the uncommon sites in addition to the bullous vesicles might be considered as having viral exanthems, leading to underrepresentation among the small rashes group. (2) Children who developed skin lesions in the later phase of infection might be under-represented if they were not hospitalized, or if they were discharged early. (3) The number of patients was relatively small due to the short epidemic period. More investigations with expanded cohort sizes are required to explore the relationship between skin phenotypes and enterovirus species, to provide evidence in clinical diagnosis.

## Conclusion

xCoxsackievirus A6 infections may cause atypical presentation of HFMD, including small vesicles on faces or vesiculobullous lesions on trunks. The majority of patients were self-limited. The pathogenesis of CVA6-induced bullae should be further investigated.

### Patients and methods

#### Definition of atypical HFMD

The definition of atypical HFMD includes symptoms of acute viral infection (such as fevers, coughs or diarrhea) with either of the following presentations: (1) maculopapular rashes presenting on the trunks, buttocks or facial areas, or (2) large vesicles or bullae on any sites of the body. Patients were classified into two groups according to vesicle sizes by two pediatricians at different points in time. The large vesicle group was defined as having vesciculobullous lesions ≥ 1 cm in diameter; the small rashes group had maculopapular rashes < 1cm in diameter. We compared the clinical symptoms between the two groups.

### Inclusion/exclusion criteria

National Taiwan University Hospital is a tertiary medical center in Taipei, Taiwan, with 266 beds in pediatric departments. Children with atypical HFMD were enrolled prospectively through the outpatient clinics and the emergency department between March and December 2010. Children who met the following conditions were enrolled: (1) Under 18 years old, and (2) skin lesions compatible with atypical HFMD. The early presentations of atypical HFMD show two distinct characteristics: (1) bullous lesions on limbs (i.e. elbows and knees), buttocks and trunks, and (2) maculopapular rashes on trunks, earlobes, and facial areas (i.e. periocular region). Compared to these features, children with typical HFMD commonly develop small vesicles (<0.5cm in diameter) or macular rashes on the knees, elbows, palms and soles. Written informed consent was obtained from each of the subject’s parents or guardians, and additional informed consent was obtained from the subject himself or herself if the subject was older than 8 years old. Pictures of specific skin lesions were taken also with consent.

Patients who met any of the following condition were excluded: (1) Failure to obtain complete demographic data, consent, or adequate clinical samples, (2) skin lesions were the results of diseases other than atypical HFMD (such as drug hypersensitivity, Kawasaki disease, or varicella infections), as determined after evaluation by related specialists, and (3) having a history of systemic illness, which might lead to an immunocompromised state (e.g. patient received chemotherapy or immunosuppressive medications).

Information regarding the demographic data, medical history and contact history were obtained from the patients or their caregivers. For hospitalized patients, our clinical observation continued in the outpatient clinic until one week after discharge, to determine any sequelae development. Two throat swabs were collected at the time of diagnosis, one for viral isolation and the other for polymerase chain reactions (PCR), to determine the etiology. Clinical symptoms and signs were recorded on the day of enrollment.

### Viral isolation

Six types of cell lines were prepared for throat swab inoculation, including human embryonic lung cells (HEL), human larynx carcinoma cells (HEp-2), rhabdomyosarcoma cells (RD), monkey kidney cells (MK-2), and Madine-Darby canine kidney cells (MDCK). After inoculating throat swabs into the cells above, daily observation of the cytopathic effect (CPE) was continued for 4 weeks. An immunofluorescence study (Chemicon International Inc., Temecula, CA) was performed once CPE occurred, to determine the pathogen.

### Polymerase chain reaction (PCR)

#### (a) viral RNA extraction

Throat swabs were prepared for viral RNA extraction, by using a MagNA Pure LC Total Nucleic Acid Isolation kit (RNA and DNA extraction kit, Roche).

#### (b) reverse transcription polymerase chain reaction (RT-PCR)

Reverse transcription was performed with a First Strand cDNA Synthesis Kit for RT-PCR (Invitrogen, CA, USA). Previously extracted viral RNA was mixed with dNTP and random hexamers. We performed real-time PCR for pan-enterovirus with primers and probes based on the highly conserved region in the 5’-untranslated region of the enterovirus genome. The sequences of primers and probes are listed as follows: forward primer, 5’-TCCTCCGGCCCCTGAATG-3’; reverse primer, 5’-AATTGTCACCATAAGCAGCCA-3’; Pan-enterovirus probe, 6FAM-AACCGACTACTTTGGGTGTCCGTGTTTCXT-PH.

The mixture was incubated for the reaction at 65°C for 5 minutes. We subsequently added reagents into the mixture, which included buffers, MgCl_2_, dithiothreitol (DTT), RNase inhibitors and reverse transcriptase. Subsequently, the samples were incubated at 25°C for 10 minutes followed by 50°C for 50 minutes, 85°C for 5 minutes, and 4°C for 5 minutes.

#### (c) molecular typing of the circulating enterovirus during the surveillance

A molecular typing of enterovirus was performed, once the samples showed positive results for enterovirus in real time RT-PCR or cytopathic effects. Three types of genogroup-specific generate oligonucleotide primers flanking the VP1 region were designed, including EntAF TNCARGCWGCNGARACNGG, EntAR outer ANGGRTTNGTNGMWGTYTGCCA, EntAR inner GGNGGNACRWACATRTAYTG; EntBF GCNGYNGARACNGGNCACAC, EntBR outer CTNGGRTTNGTNGANGWYTGCC, EntBR inner CCNCCNGGBGGNAYRTACAT; EntCF TNACNGCNGTNGANACHGG, EntCR onter TGCCANGTRTANTCRTCCC, EntCR inner GCNCCWGGDGGNAYRTACAT. The PCR product was purified using the Gel/PCR DNA fragments extraction kit (Geneaid, Taiwan) before sequencing. Auto-sequencing with the forward primer was performed on an ABI 3730XL automatic sequencer (AppliedBiosystems, CA, USA). The serotypes of the enterovirus were determined by comparing partial VP1 sequences to the sequences in the public gene database at the National Center for Biotechnology Information.

### Phylogenetic analysis

To investigate the lineage of the circulating strains, we performed phylogenetic analysis for the partial sequence of the VP1 gene of present species and several previous CVA6 strains. The comparison of sequences was based on the partial VP1 sequences of CVA6. We selected the strains of CVA6 isolated from different countries between 2004 and 2012 to construct a phylogenetic tree: a) Taiwan, GenBank: EU908166 (2004), EU908170 (2005), JN896786 (2009), JN896796 (2009), JQ390220-JQ390221 (2010), KC297130-KC297135 (2010, strains from the current study); b) USA, AY421764 (2004); c) Finland, FJ870502- FJ870508 (2009); d) Japan, AB649286-AB649291 (2011); and e) China, JQ964234 (2012). We used Geneious 6.0.4 (Biomatters Ltd., New Zealand) to analyze the sequence and complete the diagram of phylogenetic analysis.

### Statistics

We used SPSS Ver. 19 (IBM, USA) to analyze the clinical data. Descriptive statistics were expressed as mean ± standard deviation (SD) for continuous variables. The median value was used instead when continuous variables were not normally distributed. A Student’s t-test was used to compare the continuous variables with normal distributions in two independent groups; a Mann–Whitney U test was for continuous variables without normal distributions. A *Chi* square test was performed to compare categorical variables. *P-*values less than 0.05 were considered statistically significant.

## Abbreviations

HFMD: Hand-foot-mouth disease; EV71: Enterovirus 71; CVA16: Coxsackievirus A16; CDC-Taiwan: Centers for disease control in Taiwan; CVA5: Coxsackievirus A5; CVA6: Coxsackievirus A6; PCR: Polymerase chain reaction; RT-PCR: Reverse transcription polymerase chain reaction; CPE: Cytopathic effect.

## Competing interests

All the authors declare that they have no conflicting interests.

## Authors’ contributions

WCH and LYC designed and conducted this clinical study. WCH, LYC, LMH and CYL enrolled the cases and revised the manuscript. WCH performed the analyses and completed the manuscript. ALC performed the laboratory examinations. All authors read and approved the final manuscript.
